# The Effect of Irisin as a Metabolic Regulator and Its Therapeutic Potential for Obesity

**DOI:** 10.1155/2021/6572342

**Published:** 2021-03-18

**Authors:** Hui Li, Fang Wang, Mu Yang, Jiao Sun, Yi Zhao, Dongqi Tang

**Affiliations:** ^1^Center for Gene and Immunotherapy, The Second Hospital, Cheeloo College of Medicine, Shandong University, Jinan 250033, China; ^2^Institute of Medical Sciences, The Second Hospital, Cheeloo College of Medicine, Shandong University, Jinan 250033, China

## Abstract

Obesity is a worldwide health problem due to the imbalance of energy intake and energy expenditure. Irisin, a newly identified exercise-responsive myokine, which is produced by the proteolytic cleavage of fibronectin type III domain-containing protein 5 (FNDC5), has emerged as a promising therapeutic strategy to combat obesity and obesity-related complications. Various studies in mice have shown that irisin could respond to systematic exercise training and promote white-to-brown fat transdifferentiation, but the role and function of irisin in humans are controversial. In this review, we systematically introduced and analyzed the factors that may contribute to these inconsistent results. Furthermore, we also described the potential anti-inflammatory properties of irisin under a variety of inflammatory conditions. Finally, the review discussed the existing unresolved issues and controversies about irisin, including the transcription of the irisin precursor FNDC5 gene in humans, the cleavage site of the yet unknown proteolytic enzyme that cleaves irisin from FNDC5, and the reliability of irisin levels measured with available detection methods.

## 1. Introduction

Obesity, the most common nutritional disease, has become a priority public health problem worldwide, especially in developed countries. Excess weight is associated with the development of various metabolic diseases, including diabetes mellitus, hypertension, insulin resistance, cardiovascular diseases, and increased risk of cancer, which lead to higher rates of morbidity and mortality [1–4]. Furthermore, adipocytes derived from overweight and obese individuals express high levels of several key inflammatory markers as obesity causes a chronic low-grade inflammatory state [5, 6]. Numerous studies also indicated that these inflammatory markers are associated with an increased risk of a range of obesity-associated diseases [7]. Although lifestyle interventions such as physical activity and changes in diet are the primary means to reduce body weight, they have limited effectiveness or require additional interventions for other reasons in many individuals. Therefore, to reverse these alarming trends of a high prevalence of obesity and its negative impact on the quality of life, there is an urgent need for new therapeutic strategies that will produce a sustainable loss of body weight.

Irisin was discovered by Boström et al. in 2012 and identified as an exercise-triggered myokine that is presumably cleaved from the extracellular portion of the fibronectin type III domain-containing 5 (FNDC5) by an unknown protease. Irisin subsequently circulates to fat tissue where it can induce the transition of white adipose tissue (WAT) to brown adipose tissue (BAT) and regulates energy expenditure [8]. Studies also demonstrated that irisin is not only a myokine but also an adipokine, with important autocrine and paracrine functions [9]. Importantly, Luo et al. proposed that lack of irisin was associated with a poor browning response, glucose/lipid derangement, and decreased bone mass in mice [10]. These effects improve the WAT metabolic profile and enhance whole-body energy expenditure, making irisin a potential new therapeutic target for the treatment of obesity and its complications. More recent studies have shown that irisin also plays a potential role in bone metabolism [11, 12], including improving osteoblastogenesis [13–15] and enhancing bone mass and bone mineral density (BMD) [16, 17] in many physiological and pathological conditions. The relevance of irisin in humans has also been demonstrated [18–20]. In particular, the recent identification of the irisin receptor (integrin *α*V/*β*5) on osteocytes certainly facilitates new investigations between irisin and bone health [21]. However, there are controversies and obscurities regarding physiological levels and biological effects of irisin [22, 23]. In this review, we discuss the regulation of FNDC5/irisin by exercise and the potential role that irisin may play in browning WAT and its anti-inflammatory effect in mice and humans. Moreover, we also comprehensively surveyed the current studies about FNDC5 and unsolved issues about irisin, including its expression, molecular weight, and detection method. Additional pharmacological effects/physiological functions of irisin in other tissues are not discussed in this review.

### 1.1. The Role of Exercise

Physical exercise has been used as an effective tool in the prevention and management of obesity, type 2 diabetes, cardiovascular diseases, metabolic syndrome, and its complications [24]. Most myokines are expressed by muscle contraction and thought to mediate the health benefits of exercise on the metabolism. Irisin is one of these myokines, and its link with different types of physical exercise was investigated.

It was demonstrated that exercise increases the expression of peroxisome proliferator-activated receptor-gamma coactivator-1 alpha (PGC1*α*) in muscles [25], which increase thermogenesis in brown adipose tissue by regulation of mitochondrial biogenesis, and exhibited a high production of UCP-1, the biomarker of BAT [8]. Boström et al. reported that the activation of PGC-1*α* was proposed to stimulate the expression of its downstream target FNDC5 [8]. FNDC5 is a membrane protein expressed in the brain and skeletal muscle, which proved to be cleaved by yet unknown proteolytic enzyme(s) after exercises, and a new protein (named irisin) consisting of most of the fibronectin III domain is released [8]. Therefore, they proposed that circulating irisin levels are increased in individuals engaged in exercise-induced activities and progressively reduced in those less active and sedentary [8]. Following this study, there were many reports on the expression of FNDC5/irisin upon exercise, which found similar results. Exposing animals to swimming exercise resulted in a significant increment in serum irisin levels and reduced body fat mass [26–28]. Moreover, long-term exercise significantly increased expression levels of PGC-1*α* and FNDC5 in skeletal muscles in both high-fat diets and normal diet animals, compared with sedentary controls [29]. In a human study, 25 healthy young (21 ± 1 years; 16 men, 9 women) and 28 healthy middle-aged/older adults (67 ± 8 years; 12 men, 16 women) were collected to elucidate the effects of endurance training on circulating irisin levels in different age groups. The serum irisin level was significantly increased in the middle-aged/older training group after the intervention period, which was associated with a reduction in the abdominal visceral adipose tissue area and whole-body fat mass [30]. Similarly, Timmons et al. confirmed the increase in muscle FNDC5 mRNA only in the case of highly active elderly human subjects but not younger adults compared to their sedentary counterparts [31]. A prospective and controlled clinical trial also proved that low-intensity resistance exercise significantly increased circulating irisin in elderly subjects [32]. In addition, a study confirmed that circulating irisin was associated with adiposity, glucose tolerance, and insulin resistance status in a middle-aged Chinese population [33]. These results implied that the age of individuals seems to be important for changes in circulating levels of irisin. Another two studies investigated the effect of high-intensity exercise on irisin secretion and showed a significant increase in irisin concentration in individuals who underwent high-intensity exercise compared to preexercise levels [34, 35]. In addition to high-intensity exercise, Kim et al. showed that resistance exercise training caused a significant increase in circulating irisin in both mice and humans [36]. The same results were found by Lee et al. in that both resistance and endurance exercise were able to induce irisin secretion, though the highest peak was reported following endurance exercise [37]. Amanat et al. also demonstrated that 12 weeks of aerobic or aerobic combined with resistance exercise resulted in an increase in serum irisin levels [38]. However, only resistance exercise is able to promote the expression of irisin which was also proved by other studies [39, 40]. Further studies have shown that irisin injection can result in exercise-mimicking effects on metabolic parameters related to obesity, such as the concentration of adipokines, BMP4, insulin, and ghrelin [41].

Despite the huge data supporting the correlation between FNDC5/irisin and exercise, a number of studies had contradictory findings opposing the previous results in both animals and humans. Brenmoehl et al. observed that the irisin expression level was increased in skeletal muscles and serum after one bout of treadmill exercise but without an accompanying change in FNDC5 mRNA levels [42]. In addition, studies also failed to detect an association between levels of irisin or FNDC5 and exercise in rats after exercise training [43, 44]. In addition to animal studies, human studies also failed to detect the correlation between PGC-1*α* or FNDC5/irisin and exercise. Moreover, contradictory results were obtained from different laboratories where the activation of PGC-1*α* and FNDC5 expressions was not coupled to skeletal muscles during exercise. Raschke et al. conducted experiments to evaluate the expression of FNDC5 using an in vitro contraction electrical pulse stimulation (EPS) model in human primary skeletal muscle cells. However, they discovered that although PGC-1*α* mRNA expression was significantly enhanced, FNDC5 mRNA expression remained unchanged. Similar results were also obtained from two different training cohorts. Neither 10 weeks of interval endurance training (41 ± 2 years old males) nor 11 weeks of strength training in healthy men (28 ± 4 years old males) resulted in increased FNDC5 mRNA expression in skeletal muscle biopsies [45]. This was the first study questioning the existence and importance of irisin in humans. Later, Kurdiova et al. reported that exercise-mimicking treatment with forskolin and ionomycin in human primary muscle cell cultures stimulated the expression of PGC-1*α* but decreased the expression of FNDC5 and irisin secretion [46]. A meta-analysis including 51 studies reported that a solid conclusion about the link between PGC-1*α* activity and FNDC5 expression in response to physical activity could not be made [47]. Therefore, the upstream regulatory effect of PGC-1*α* on the FNDC5 gene needs to be further confirmed in humans. In line with these findings, other clinical studies also did not confirm exercise-related irisin regulation [48–50]. Although Norheim and his group observed a significantly increased PGC-1*α* and FNDC5 mRNA expression in 26 individuals (40–65 years) after 12 weeks of chronic resistance and strength exercises, circulating levels of irisin were paradoxically reduced [51]. This is in agreement with Park's studies where regular exercise was inversely correlated with irisin levels in adult men [52]. These inconsistent changes in PGC-1*α*, FNDC5, and circulating irisin levels suggest that other unknown factors such as ATP homeostasis described by Huh et al. may be involved in the regulation of exercise-induced irisin effects [53]. In addition, the regulation effect of exercise on irisin under abnormal metabolic conditions was also studied and showed conflicting results in different exercise protocols [42, 54]. Thus, the molecular mechanisms of exercise-regulated PGC-1*α*/FNDC5/irisin signaling are still far from being clear.

The current data lead us to propose that the following factors may contribute to the inconsistent results. Until now, the most commonly used method for detecting circulating irisin levels was based on commercially available ELISA kits that have questionable validation. In addition, subjects' age, sex, and conditioning status (such as renal failure and hormonal conditions) may also be critical factors. As described by Scalzo et al. when measuring changes in the calculation of irisin and expression of the FNDC5 gene after nine high-intensity interval training sessions over a three-week period, opposite associations were found in women and men [55]. However, it has also been reported that there were no training-induced (sex-specific) changes in circulating irisin levels in Type 2 Diabetes Mellitus (T2DM) patients [56]. Moreover, the discrepancy in the results can also be explained by the time frame considered for the evaluation of irisin levels and the variation in the protocols of exercise. For example, studies that tested irisin at various times before and after exercise raised the hypothesis that irisin levels increase for a limited period of time after exercise and do not continue to remain elevated [57]. Furthermore, studies that were used for irisin determination were based on either fresh or frozen samples. However, Hecksteden et al. observed that irisin is prone to storage-related degradation [48]. Therefore, time-related changes in circulating irisin concentrations in the absence of timed-matched controls should be interpreted with caution. Also, other factors that are believed to regulate the plasma levels of irisin have been identified such as cold exposure, obesity [58], the glucose and lipid profile [59], and myostatin [60].

It is worth noting that physical exercise improves the quality of life and reduces the incidence of several disorders through various molecular pathways and myokines, but FNDC5/irisin may not be the only factor involved in this process. Many genes are activated in human skeletal muscle by exercise training [61, 62], all of which may contribute to improving health. Therefore, further work is necessary to comprehensively consider the association between irisin and exercise and its impact on human health.

### 1.2. Impact of Irisin on Adipose Tissue Browning

#### 1.2.1. Types of Adipose Tissue

Adipose tissue is a highly complex and heterogeneous tissue with many physiological and pathological roles. WAT and BAT are two typical adipose tissues derived from different lineages and having inverse functions [63]. The main function of WAT is to store energy in the form of triglycerides, while BAT can dissipate energy as heat through mitochondrial uncoupled respiration [64, 65]. In recent years, the third type of thermogenic cell formation from white adipocytes with the capability to increase thermogenesis was described and termed “brite” (brown in white) adipocytes or beige adipocytes [66–69]. These inducible brite adipocytes are distinct from classical brown adipocytes but share several biochemical features such as increased UCP1 gene expression and the ability to dissipate energy through a thermogenic response. UCP-1 exists in the mitochondrial endomembrane and uncouples electron transport from ATP production [70]. Arhire et al. systematically reviewed the characteristics and thermogenesis of different adipose tissues [71]. The advantage of browning, compared to classical BAT, is that adult humans have very little BAT with a minimal energy-wasting potential, but an abundance of WAT that has the potential to brown, which could produce a much more dramatic energy expenditure. Therefore, the enrichment and activation of beige adipocytes represent an attractive therapeutic strategy to combat obesity and obesity-related complications [72]. Accumulating evidence indicates that many hormones and cytokines can promote lipid metabolism and increase energy expenditure through autocrine or endocrine mechanisms [73]. Among them, irisin is the adipomyokine of great hope for increasing energy expenditure and regulating thermogenesis [8]. In addition to irisin, parathyroid hormone (PTH) is also considered an effective transcriptional mediator for regulating the thermogenic program in white or brown adipocytes [74, 75]. Moreover, PTH has been shown to have several metabolic effects that appear to oppose those of irisin [76, 77]. In particular, the recent preclinical finding suggests the existence of an interplay between PTH and irisin metabolism [78]. Therefore, irisin with its potential to induce the browning of white adipocytes and activate metabolism has attracted much attention in this field.

#### 1.2.2. Studies in Mice

Boström et al. reported that irisin with its precursor FNDC5 plays a major role in browning white adipose tissue and activation of thermogenic genes. After primary murine subcutaneous fat-derived preadipocytes were treated with 20 nM commercial FNDC5 protein during adipogenic differentiation, expressions of the uncoupling protein UCP1 and other BAT-related genes were increased. By contrast, FNDC5 failed to enhance brown marker genes in classical brown adipocytes isolated from the interscapular depot, suggesting the depot-specific effects for FNDC5. Also, they demonstrated the beneficial metabolic regulation effect of irisin in vivo, as 20-week high-fat diet-induced obesity was reduced by adenoviral-mediated overexpression of FNDC5 in mice [8]. Wu et al. identified a distinct pool of progenitors within WAT that can give rise to beige cells that are similar but not identical to classical brown fat cells. In their study, CD137-high expressing cells display a strong browning response toward irisin and FNDC5 compared to CD137-low expressing cells [67]. These data suggest that irisin might have subtle effects on the subpopulation of preadipocytes isolated from the subcutaneous depot, which highly express CD137. Later, our group demonstrated that irisin can potentially prevent obesity and associated type 2 diabetes by stimulating the expression of WAT browning-specific genes. Moreover, we found that the browning effect induced by irisin was mediated by p38 MAP kinase and ERK MAP kinase signaling [79]. In addition, irisin has also been shown to exert its browning and other essential functions through additional pathways [80, 81].

#### 1.2.3. Studies in Humans

Although it has been demonstrated that irisin plays a pivotal role in inducing fat browning and regulation of energy expenditure in animal studies [8, 79, 82], the function of irisin in humans remains to be elucidated. If these findings in mice could be translated to humans, irisin could be a promising therapeutic agent for the treatment of obesity. However, studies investigating the function of FNDC5/irisin in humans are still rare, and it remains controversial whether results about browning obtained in murine models can be extrapolated to humans. Raschke et al. showed evidence against a beneficial effect of irisin in humans in that neither recombinant FNDC5 nor irisin triggered a brite differentiation of primary human preadipocytes isolated from the subcutaneous depot [45]. Moreover, they also demonstrated that high CD137 expression in human subcutaneous adipose tissue was not positively correlated with the browning effect of FNDC5/irisin, which is inconsistent with Wu's results [67]. Lee et al. showed that FNDC5 enhanced a BAT-like thermogenic program in primary human adipocytes isolated from neck biopsies and to a lesser extent subcutaneous adipocytes but was completely absent on omental adipocytes [37]. In addition, Huh et al. investigated the potency of irisin on preadipocyte differentiation and discovered a significant inhibitory effect of irisin on both human and mouse preadipocyte differentiation, whereas the expression of genes and/or proteins related to browning remained unaffected [83]. This is in contrast to the browning effect of human adipocytes after incubation with bone morphogenetic protein 7 (BMP7) [45], and this factor was reported to accelerate preadipocyte differentiation [84]. To further explore the contradictory browning effects of irisin on human cell models, our group examined the longitudinal effects of irisin during adipogenic differentiation on different donors. In line with the study by Huh et al. [83], human preadipocytes derived from subcutaneous adipose tissue demonstrated a decrease in differentiation to mature adipocytes after irisin treatment, whereas the expression of genes and/or proteins related to browning (for example, UCP1, PPAR*γ*, and PRDM16) was also decreased. By contrast, in mature human adipocytes, irisin stimulates browning, indicated by the upregulation of browning related genes (UCP1, PGC-1*α*, and PRDM16), and this action was mediated by activating the ERK and p38 MAPK signaling pathways [85]. Furthermore, irisin-treated BAT from perirenal fat showed no further activation of p38/ERK MAPK signaling or expression of browning-related genes was found in this study. A large body of evidence indicates that the accumulation of visceral white adipose tissue (vcWAT) is more pathogenic than subcutaneous white adipose tissue (scWAT), as the former carries a greater risk of developing obesity, cardiovascular events, atherosclerosis, hypertension, and other metabolic diseases [86, 87]. Therefore, to systematically examine the effects of irisin on human visceral adipose tissue and adipocytes is critical to further understand its molecular and biological functional properties. Through further research, our group found that irisin exerts an inhibitory effect on lipid accumulation during vcWA- derived preadipocyte differentiation [88]. However, in contrast to subcutaneous adipocytes, expressions of UCP-1 and other brown-related genes were not induced in irisin-treated differentiated mature visceral adipocytes. Notably, irisin increased mitochondrial metabolism in all three types (scWAT, vcWAT, and BAT) of adipose tissue-derived primary cultured adipocytes. This is surprising as the thermogenic capability of brown fat is mainly mediated by mitochondrial protein UCP-1, which uncouples the electron transport chain from energy production, resulting in the release of energy as heat. The reasons why the fat depot specificity action of irisin on UCP-1 did not translate into its differences in mitochondrial respiration are unknown but might involve interactions with other factors modulating the complex mitochondrial biogenesis program.

In conclusion, these data suggested that contradictory findings still exist in humans and mouse studies, and several reasons may account for these controversies. First, irisin's browning effect on humans is only observed after the formation of mature adipocytes, which may at least partly explain the conflicting reports of irisin's effects on human adipocytes. It is not hard to understand that the epigenetic change in human primary mature adipocytes involves the dedifferentiation and redifferentiation process in vitro [89]. Moreover, the loss of other types of cells, such as endothelial cells, multipotential mesenchymal cells, nerve cells, and immune cells in fat tissue, and the lack of a 3D structure are limitations of this approach. In addition to adipocytes, many adipokines (e.g., TNF*α*, IL-6, or MCP-1) secreted by other cells exist in fat tissue [90], which can also generate signals at local and peripheral levels. It is believed that these adipokines influence many metabolic pathways as well as the differentiation of adipocytes. Second, irisin may exert differential effects depending on the location/types of the adipose tissue. Adipocytes derived from different progenitor cells exhibit different gene expression patterns and may respond differently to irisin [91]. Third, the irisin-induced thermogenic gene program is mediated by signaling through *α*V/*β*5 integrin [21], as recently reported by Kim et al. Therefore, the expression of this receptor may differ between various types of adipocytes, which result in different responses to irisin.

### 1.3. Anti-Inflammatory Effects of Irisin

Obesity causes a chronic low-grade inflammatory state accompanied by proinflammatory macrophage infiltration into white adipose tissue, which is associated with the development of insulin resistance, and increased risk of cardiovascular disease [5, 6]. Moreover, obese adipose tissue per se expresses numerous genes which are involved in inflammatory pathways [92]. The potential protective effect of irisin on obesity-related diseases may be partly attributed to the anti-inflammatory properties of irisin by activating various signaling pathways [81]. Mazur-Bialy et al. published three important papers studying the anti-inflammatory effects of irisin on adipocytes [93] and macrophages [94, 95]. They initially found that irisin markedly changes macrophage activity, improves their ability for phagocytosis, and reduces the intensification of processes connected with ROS production, which could suggest its potential anti-inflammatory properties [94]. Subsequently, they confirmed that irisin exerts its potential anti-inflammatory properties in RAW 264.7 macrophages through the downregulation of downstream pathways of TLR4/MyD88 [95]. Moreover, they also demonstrated for the first time that irisin can directly attenuate the inflammation process in lipopolysaccharide (LPS) activated cultured adipocytes by suppressing the expression of proinflammatory cytokines [93]. Later, the same authors conducted a coculture system of 3T3 adipocytes and RAW 264.7 macrophages and showed that both glycosylated irisin and nonglycosylated irisin effectively inhibit the expression and release of inflammatory mediators, although nonglycosylated irisin has a stronger anti-inflammatory potential [96].

Macrophage infiltration in WAT is associated with obesity causing a phenotypic switch in these cells from an anti-inflammatory M2 to a proinflammatory M1 state [97]. Dong et al. demonstrated that irisin can reverse this process by stimulating macrophage polarization from M1 to M2 types [60]. In addition, irisin has been shown to reduce the expression of TNF-*α*, IL-6, MCP-1*α*, and MIP-1*α*, while enhancing the expression of IL-10 in human visceral and subcutaneous fat tissue [88]. Interestingly, recent research found that traditional Chinese treatment electrical auricular acupuncture (EAA) could also reduce body weight and suppress inflammation through promoting norepinephrine (NE) release from the adrenal gland leading to further expression of FNDC5, irisin, and UCP-1 [98]. In addition to adipocytes and macrophages, irisin is also involved in the anti-inflammatory effects of other tissues and organs [99–111], as shown in Table 1.

Both animal and in vitro studies suggested the potential anti-inflammatory effects of irisin by modulating the production of cytokines, influencing transcription factors as MAPK and nuclear factor-kappa B, or reducing the production of reactive oxygen species. Nevertheless, studies in this field are still rare, and further mechanistic studies of the effects of irisin on inflammation are needed to provide additional insights.

### 1.4. The Discoveries, Structure, and Function of FNDC5

FNDC5 was first discovered by two groups in a genomic search for fibronectin type III domains in 2002 [112]. FNDC5 is a transmembrane protein including a signal peptide, two fibronectin domains, and one hydrophobic domain inserted in the cell membrane. A previous study suggested that FNDC5 is located in the matrix of peroxisomes, because when they expressed FNDC5 with a green fluorescent protein (GFP) fused to its N terminus, a punctate localization to peroxisomes was found [113]. In 2012, Boström et al. questioned the studies of Ferrer-Martinez et al. and considered that FNDC5 might be a secreted protein and described irisin as a cleaved and secreted part of the transmembrane protein FNDC5 [8]. Later, Erickson also provided some evidence to refute that FNDC5 is a peroxisomal protein [22]. In humans, three FNDC5 variants have been identified and these three FNDC5 variant genes are distributed in various human tissues including the heart, brain, liver, skeletal muscles, pancreas, and ovaries and have different expression levels [114]. However, the existence of other FNDC5 transcripts was also confirmed in humans [115]. Currently, most studies pay relatively more attention to irisin, while ignoring the important role of its precursor FNDC5 in the regulation of energy metabolism. The available literature clearly indicates FNDC5 participation in maintaining the metabolic homeostasis of the body under different physiological or pathophysiological conditions, while FNDC5 dysregulation may lead to systemic metabolism imbalance and eventually result in the onset of metabolic disorders [116–120]. As described by several studies, mRNA and protein levels of FNDC5 increased in muscle tissues of obese mice induced with a high-fat diet (HFD) or in obese/diabetic-prone Otsuka Long Evans Tokushima Fatty rats compared with lean/healthy controls [121–123]. Other studies also showed that FNDC5 overexpression in obese mice induced with HFD increases energy expenditure, attenuates hyperglycemia and insulin resistance, and activates lipolysis in adipose tissues [82]. In addition to obesity-related metabolic diseases, obesity-induced chronic inflammation is also critical in the pathogenesis of insulin resistance. Xiong et al. showed that FNDC5 prevents HFD-induced obesity, insulin resistance, fat accumulation, and inflammation through the downstream mediator AMPK signaling pathway [124]. Another group of researchers reported a positive correlation between FNDC5 expression and anti-inflammatory cytokine IL-10 and a negative correlation with TNF-*α* levels [125]. These results not only imply a potential regulatory mechanism of FNDC5 to offset a high-fat diet-induced weight gain by increasing energy expenditure but also show the potential to be used as a therapeutic regimen for preventing inflammation and insulin resistance in obesity and diabetes. In addition to its therapeutic targets for obesity-associated maladies, FNDC5 is also involved in other metabolic diseases. Liu et al. observed that FNDC5 deficiency exacerbates whereas FNDC5 overexpression prevents HFD-induced hyperlipemia, hepatic lipid accumulation, and impaired fatty acid oxidation (FAO) and autophagy in the liver via the AMPK/mTOR pathway [126]. In addition, FNDC5 has been demonstrated to attenuate obesity-induced cardiac hypertrophy by inactivating JAK2/STAT3 associated-cardiac inflammation and oxidative stress [127]. The systematic review by Zhang et al. also summarizes its biological functions in a variety of metabolic diseases [128, 129].

The activation and transcriptional regulation of FNDC5 have been poorly studied, and although some studies have determined associations, no direct activator of FNDC5 has been identified. Tiano et al. revealed that, compared with wild-type mice, exercise increased serum irisin and skeletal muscle FNDC5 and its upstream PGC-1*α* expression in SMAD3-deficient mice. Moreover, through in vitro experiments in myotubes, they further demonstrated that SMAD3 inhibits FNDC5 and PGC-1*α* expression in skeletal muscle cells by binding to their promoters [130]. In another study, it was reported that leptin can also reduce FNDC5 mRNA expression in subcutaneous adipose tissue from nonobese subjects [131]. On the other hand, studies also proved that FNDC5 can be activated by some upstream signaling molecules. Yang et al. observed that CREB overexpressed C2C12 myotubes display higher FNDC5 expression and further proved that PGC-1*α*/CREB interaction triggers this effect [132].

In general, literature mining indicates that FNDC5 not only plays a vital role in energy metabolism but also has crucial roles in a variety of processes such as inflammation, autophagy, and oxidative stress. Therefore, fully understanding the biological function and precise underlying mechanisms of FNDC5 is of the same importance as irisin.

### 1.5. Existing Controversy

**Figure 1:**
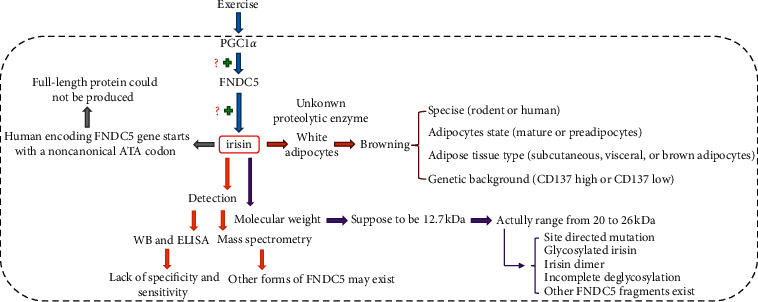
The existing controversies about irisin.

Following the discovery of irisin, researchers have shown much interest because of its potential use as a therapeutic agent in the treatment of metabolic and endocrine disorders. After many years of research, however, the proteolytic enzyme that cleaves irisin from FNDC5 has yet to be identified [133]. Thus, further studies are necessary to determine if there are different secretory mechanisms in addition to proteolysis [8]. Additionally, the putative myokine irisin has been the subject of debate since its initial description [134, 135]. In addition to inconsistencies regarding the regulation of FNDC5/irisin by exercise and the effect of irisin on white fat browning, there has been great conflict regarding its expression and detection (Figure 1).

First, in humans, the FNDC5 gene has three variants that are distinguished by the signal peptide and C-terminal amino acids. Transcript 1 represents the longest transcript and produces a truncated FNDC5 protein from an in-frame ATG codon in exon 3, and the proposed irisin peptide lacks the first 44 amino acids. Transcripts 2 and 3 that initiate translation from an atypical ATA have been proved to have a lower translational efficiency (only 1% of full-length FNDC5) as compared to the typical ATG start codon [45]. The usage of the downstream canonical ATG as an alternative start site leads to the expression of a truncated protein containing only parts of the proposed irisin peptide. However, several lines of evidence stand against this claim. As demonstrated by some studies, many eukaryotic encoding genes start with different non-ATG codons, and all of them produce complete and functioning proteins [136–138]. Moreover, Jedrychowski et al. ascertained in their initial studies that irisin circulates in its full-length form and that its production originates from the noncanonical ATA codon [34]. In Albrecht's study, evidence was provided that one or more FNDC5 transcripts exist in human skeletal muscles, and they all are likely to be translated from a noncanonical ATA codon [115]. To date, there is no clear evidence of the transcription of human FNDC5 from either a canonical or noncanonical start codon, and more studies are necessary to determine what are the precise roles of different forms of FNDC5.

Second, in the initial report, irisin was proposed to be cleaved from its precursor FNDC5 and the theoretical molecular weight was 12.7 kDa [8]. However, many studies detected irisin with a molecular weight ranging from 20 to 26 kDa in serum or plasma of different species, which is more likely to reflect full-length FNDC5 without its signal peptide (glycosylation: ∼27 kDa; deglycosylation: ∼20 kDa) [9, 37, 139, 140]. Although mass spectrometry found that these bands contain a unique sequence of irisin, the possibility of other FNDC5 fragments cannot be ruled out [37]. Albrecht et al. found a greater transcript diversity of human FNDC5 than the currently annotated one [115]. In Jedrychowski's study, this inconsistency was attributed to incomplete deglycosylation of irisin with PNGase F, because a 12 kDa irisin peptide could be detected in human plasma after the removal of albumin and IgG, and deglycosylation by using Protein Deglycosylation Mix from NEB [34]. However, in another study, the same method was used but no human irisin was detected in plasma [141]. Actually, no differences in the efficiency of deglycosylation of recombinant irisin between PNGase F and the Protein Deglycosylation Mix (NEB) were observed in Albrecht's study [115], and no specific bands for irisin were detected in human serum samples incubated with either PNGase F or the deglycosylation mix. Other explanations for the inconsistent results in circulating irisin detection may be due to site-directed mutation (16 kDa), irisin dimer (23.5 kDa), and glycosylated irisin (36 kDa). Therefore, the cleavage site of the FNDC5 by an unknown protease and the existence of other soluble FNDC5 isoforms needs to be further studied.

Third, a number of the studies that quantitated the level of irisin in plasma were largely based on commercially available antibodies or ELISA kits. However, these kits were proved to have questionable validation, as different studies using different kits measured irisin levels in a wide range from picograms to micrograms per milliliter of serum or plasma [142–147]. Moreover, the lack of specificity and sensitivity is the main disadvantage of this method, which compromises reliable measurements of irisin with ELISAs. Albrecht et al. tested several polyclonal antibody-based ELISA kits and showed that these antibodies had prominent cross-reactions with nonirisin proteins in serum or plasma of different species [23]. In addition, they found that an FNDC5 signature was identified by mass spectrometry in human serum samples, but it was not detected by commercial ELISA irisin kits. Therefore, the measurement of circulating irisin is still challenging, and the reliability of irisin antibodies needs to be further proven.

## 2. Conclusions and Future Prospects

Notwithstanding the fact that the expressed form of irisin remains unclear in humans, it has been the subject of many studies due to its proposed therapeutic potential for the treatment of obesity and obesity-associated maladies through browning of WAT. Studies also showed that irisin is involved in mediating several other exercise-inducible beneficial effects on health, such as improving energy consumption, glucose utilization, and insulin resistance. Surprisingly, a recent study demonstrated that irisin also presented a very positive effect on the regulation of diverse genes related to the COVID-19 outcome in the human subcutaneous adipose tissue [148]. However, the mechanisms underlying these benefits are unclear, in large measure, because some issues about irisin have yet to be solved. Recently, Kim et al. demonstrated that irisin binds directly to *α*V integrin receptors to promote osteocyte survival and indicated that irisin induces a thermogenic program in fat also via binding to the same receptors [21]. This gained a lot of importance in understanding the molecular mechanisms underlying the beneficial role of irisin in various physiological conditions and disease states. However, as integrin receptors are widely expressed on various cell surfaces in vivo, the possibility of other irisin-specific receptors may exist to regulate its activity. In addition, whether the physiological activity of irisin in other tissues and organs is also realized through integrin receptors remains to be further investigated.

In conclusion, although studies on physiological functions and circulating levels of irisin have shown much controversy in humans, FNDC5/irisin has raised great expectations as a potential target in the conservative treatment of obesity. Further studies are certainly needed to clarify the conflicting and obscured results obtained in humans, which requires the establishment of reliable technical assays for quantifying circulating irisin levels.

## Figures and Tables

**Table 1:** Summarizing the anti-inflammatory effects of irisin in various inflammation models.

Study (reference)	Intervention	Main findings
Adipocytes [93]	3T3-L1 cells stimulated by LPS	TNF-*α*, IL-6, MCP-1, and NF-*κ*B↓
Macrophages [95]	RAW 264.7 cells stimulated by LPS	IL-1*β*, TNF-*α*, IL-6, MCP-1, KC, HMGB1, and NF-*κ*B↓
Adipocytes and macrophages [96]	3T3 adipocytes and RAW 264.7 cells coculture and stimulated by LPS	IL-1*β*, TNF*α*, IL-6, MCP-1, and HMGB1↓
Peritoneal macrophages [60]	Mouse peritoneal macrophages stimulated by LPS	Phenotypic switching of peritoneal macrophages from M1 ⟶ M2
INS-1E *β* cells [108]	INS-1E *β* cells stimulated by glucolipotoxic conditions	COX2, CCL2, CXCL1, and NF-*κ*B↓

Clinical disease		
Atherosclerotic [100]	HFD Apolipoprotein E-deficient mice and carotid partial ligation mouse model	IL-6, MCP-1, ICAM-1, VCAM-1, and NF-*κ*B↓
Ali [99]	LPS-induced acute lung injury mouse model; LPS-induced A549 cells	IL-1*β*, IL-6, MCP-1, TNF-*α*, MAPK, and NF-*κ*B↓
IBDs [105, 106]	TNBS-induced IBD rats	Gut and osteocyte proinflammatory cytokines↓; bone formation↑, osteoclast surface↓
	DSS-induced IBD rats	Improved colon inflammation; bone formation rate↑, osteoclast surface, and osteocyte proinflammatory factors↓
Hepatic steatosis [107]	PA-induced AML12 cells and mouse primary hepatocytes	NF-*κ*B, COX-2, p38 MAPK, TNF-*α*, IL-6, and PRMT3↓
Liver injury [111]	LPS-mediated liver injury	IL-6, IL-1*β*, TNF-*α*, and NF-*κ*B↓

LPS, lipopolysaccharide; TNF-*α*, tumor necrosis factor *α*; IL-6, interleukin 6; MCP-1, monocyte chemotactic protein 1; NF-*κ*B, nuclear factor-*κ*B; IL-1*β*, interleukin 1*β*; KC, keratinocyte chemoattractant; HMGB1, high mobility group box 1; COX2, cyclooxygenase-2; CCL2, chemokine (C-C motif) ligand 2; CXCL1, chemokine (C-X-C motif) ligand 1; ICAM-1, intercellular cell adhesion molecule-1; VCAM-1, vascular cell adhesion protein 1; ALI, acute lung injury; MAPK, mitogen-activated protein kinase; IBDs, inflammatory bowel diseases; TNBS, 2,4,6-trinitrobenzenesulfonic acid; DSS, dextran sodium sulfate; PA, palmitic acid; PRMT3, protein arginine methyltransferase-3.
